# Curcumin-Loaded BSA Nanoparticles Protect More Efficiently Than Natural Curcumin Against Scopolamine-Induced Memory Retrieval Deficit

**DOI:** 10.32598/bcn.9.10.255

**Published:** 2019-03-01

**Authors:** Roksana SoukhakLari, Leila Moezi, Fatema Pirsalami, Morteza Abkar, Maryam Moosavi

**Affiliations:** 1. Shiraz Neuroscience Research Center, Shiraz University of Medical Sciences, Shiraz, Iran.; 2. Student Research Committee, School of Medicine, Shiraz University of Medical Sciences, Shiraz, Iran.; 3. Nanobiology and Nanomedicine Research Centre, Shiraz University of Medical Sciences, Shiraz, Iran.; 4. Department of Pharmacology, School of Medicine, Shiraz University of Medical Sciences, Shiraz, Iran.

**Keywords:** Curcumin, Albumin, Memory, Nanotechnology, Mice

## Abstract

**Introduction:**

There is evidence indicating that the rate of AD is lower in curry consuming populations. Then, there is an effort to elucidate if curcumin -as the main ingredient of turmeric-might affect the process of AD. However, in clinical trials of AD, a six-month curcumin treatment failed to show any progress, which might be attributable to its low bioavailability. In this line, a recent human study revealed that a more bioavailable solid lipid curcumin enhances cognition in aged adults. By the application of Bovine Serum Albumin (BSA), the current study aimed at converting curcumin to nano sizes and assessing its protective effects against scopolamine-induced passive avoidance memory retrieval deficit.

**Methods:**

Nanocurcumin was prepared via dissolution method. Male NMRI mice (20–25 g body weight) were used. The effective doses of nanocurcumin were selected according to the initial pilot test. The mice were treated with nanocurcumin 15 or 20 mg/kg/p.o or distilled water for 10 days. The animals were habituated and trained in passive avoidance apparatus on the day 10. The retention test was performed 24 hours later. Scopolamine (1 mg/kg/i.p.) or saline was injected 30 minutes before memory retention trial.

**Results:**

The findings indicated that nanocurcumin in doses 15 or 20 mg/kg/p.o prevented the retrieval deficit induced by scopolamine while natural curcumin in its equivalent doses did not have such an effect. Furthermore, nanocurcumin by itself improved memory retention comparing with the control group.

**Conclusion:**

These findings implied that the potential anti-amnesic effects of curcumin might be observed by producing and using its nanoformulation form.

## Highlights

- Bovine Serum Albumin (BSA)-based nanocurcumin prevents scopolamine-induced memory retrieval deficit.
- Oral BSA-based nanocurcumin improves memory in adult mice.
- The equivalent doses of natural curcumin have no effect on scopolamine-induced amnesia.

## Plain Language Summary

Some population studies show that India has the world’s lowest rate of Alzheimer’s Disease (AD), perhaps because their traditional diet involves turmeric in almost every meal. Curcumin is the main compound found in turmeric. In AD patients, a 6-month curcumin treatment failed to show any progress which might be attributable to the low bioavailability properties of curcumin. In this line, a recent human study showed that a more bioavailable solid lipid curcumin improves cognition in aged adults. Using BSA (bovine serum albumin), this study aimed to break curcumin to nanosize particles to increase its bioavailability and assess its effect on memory. Male adult mice were treated with nanocurcumin. Following this treatment period, behavioral studies were performed. The findings indicated that nanocurcumin prevented the retrieval deficit induced by scopolamine (a drug which induces amnesia) while natural curcumin in its equivalent doses did not have such an effect. Furthermore, nanocurcumin by itself improved memory compared to the control group. These findings imply that curcumin has potential memory improving the effect and might combat aging-induced amnesia.

## 1. Introduction

The lower prevalence of Alzheimer’s Disease (AD) in curry consuming populations suggests its protective role against brain dementia (Ganguli et al., 2000; Ng et al., 2006). Therefore, there is a trend to find if curcumin, as the main ingredient of turmeric, has a protective role in patients with AD. However, the research works conducted on patients with AD show no difference between patients treated with curcumin and placebo (Baum et al., 2008; Ringman et al., 2012). This lack of effect is supposed to be related to the low bioavailability of curcumin (Anand, Kunnumakkara, Newman, & Aggarwal, 2007; Witkin et al., 2013); since a solid lipid curcumin with higher bioavailability is reported to boost cognitive function and mood in non-demented adult humans (Cox, Pipingas, & Scholey, 2015).

According to the cholinergic deficit observed in AD (Bartus, Dean, Beer, & Lippa, 1982; Tohgi, Abe, Kimura, Saheki, & Takahashi, 1996), “the cholinergic hypothesis” of AD was introduced (Bartus, Dean, & Beer, 1982) and thereby, the inhibitors of acetylcholine esterase such as tacrine and donepezil were assumed as one of the medical treatments of AD (Lahiri, Rogers, Greig, & Sambamurti, 2004). Scopolamine, as the muscarinic receptor antagonist, is therefore used to induce a pharmacologic animal model of amnesia (Moosavi, Khales, Abbasi, Zarifkar, & Rastegar, 2012; Sarter & Bruno, 1997; Sunderland et al., 1986).

Memory is regarded as a three-step process: acquisition, consolidation, and retrieval (Atkinson & Shiffrin, 1968). In AD, one of the most serious problems is forgetting the important well-known data such as the name of their children and spouses (Bennett, 2000). Although the amnesia observed in AD was considered to be caused by a problem in memory encoding, recently it is shown that there is a retrieval deficit than a retention problem (Roy et al., 2016). In this regard, scopolamine is found to impair memory retrieval in tasks such as passive avoidance (Moosavi, SoukhakLari, Moezi, & Pirsalami, 2017; Rush, 1988).

It was previously shown that curcumin prevented pre-training (Akinyemi, Oboh, Oyeleye, & Ogunsuyi, 2017; Barber et al., 2016; Lim et al., 2016) or post-training (Ahmed & Gilani, 2009; Ali & Arafa, 2011; Sarlak, Oryan, & Moghaddasi, 2015) scopolamine-induced amnesia, but it is not known if it prevented scopolamine-induced memory retrieval deficit. Due to the higher bioavailability of nanocurcumin and the recent findings regarding the importance of retrieval stage of memory in AD state (Roy et al., 2016), the current study aimed at formulating a Bovine Serum Albumin (BSA) based nanocurcumin (Jithan, Madhavi, Madhavi, & Prabhakar, 2011) and assessing its effect against pre-test scopolamine-induced memory retrieval deficit (Moosavi et al., 2018).

## 2. Methods

### 2.1. Animals

Male NMRI mice (50–60 days age-old) were obtained from the animal lab of Shiraz University of Medical Sciences. The animals were kept in animal house at a controlled room temperature (21±2°C) under a normal 12:12-hour light-dark cycle with free access to food and water. All experiments were approved by the Ethics Committee for Animal Experiments of Shiraz University of Medical Sciences and the animal experiments were conducted in accordance with the NIH Guide for the Care and Use of Laboratory Animals. The mice were randomly allocated to groups of eight for the main workand groups of three for the initial pilot study.

### 2.2. Materials

Curcumin was purchased from Exir Company, Iran, and Scopolamine from Sigma, USA. Other reagents were obtained from commercial sources.

### 2.3. The preparation of BSA-based nanocurcumin

BSA was used as the polymer to prepare nanocurcumin (Aniesrani Delfiya, Thangavel, & Amirtham, 2016; Jithan et al., 2011; Moosavi et al., 2018). BSA solution 3% was prepared using distilled water. Then, curcumin 0.25% (w/v based on BSA solution) was prepared in acetone.

The prepared curcumin solution was added intermittently into BSA solutions on a magnetic stirrer at 500 rpm at room temperature to achieve the cross-linkage of the dissolved BSA nanoparticles. Each time, 2 mL of curcumin solution was added in a five-minute interval to BSA solution and this process was continued until the turbidity of the solution. At that time, glutaraldehyde (110 μL of 8% in distilled water solution) was added to achieve the cross-linkage of nanoparticles.

The complete cross-linking process of this colloidal suspension was accomplished during another 24 hours in cold room. Purification of formed nanoparticles was performed by five cycles of centrifugation (3000 rpm, 30 minutes) followed by dispersing with distilled water to remove unreacted chemicals and the dissolving agents. A freeze drier was used to dry the purified pellet to achieve a powder.

### 2.4. Characterization of nanoparticles

A specimen of the acquired powder was dissolved in distilled water and prepared for Field Emission Scanning Eectron Microscopy (FESEM). Following coating the sample with gold, FESEM was accomplished using a TESCAN Mira3-XMU (Czech Republic) microscope.

### 2.5. Drug administration

As the content of curcumin in the achieved powder was ¼, the doses of curcumin itself were reported in current study. After dissolving in distilled water, nanocurcumin was administered via gavage during 10 days. Afterward, the behavioral experiments were performed to investigate the effect of nanocurcumin on passive avoidance memory retention. To know the effective dose of this nanoformulated curcumin against scopolamine, a series of pilot tests were performed and a range of doses 2.5, 5, 10, 15, 20, and 25 mg/kg/p.o. were administered to the mice in groups of three.

To induce memory deficit, scopolamine (1 mg/kg) was dissolved in saline and injected intraperitoneally 30 minutes before the test (Moosavi et al., 2018). According to the results of the performed pilot study, two effective doses (15 and 20 mg/kg) were selected for the main study.

### 2.6. Behavioral studies

#### 2.6.1. Passive avoidance apparatus

The shuttle box apparatus consisted of two equal light and dark compartments (17×12×15 cm) separated by a guillotine door (9×17 cm). This door could be raised up to 10 cm. The stainless steel floor of the apparatus was connected to a shock stimulator which could transfer the electrical shock to the floor of the dark compartment.

#### 2.6.2. Habituation

To habituate with the apparatus, the animal was placed in the light compartment on the day 10. Following a 10-second delay, the door was raised, and the animal was allowed to enter the dark compartment. Another habituation trial was performed 30 minutes later.

#### 2.6.3. Training

The training was performed 30 minutes following the second habituation trial. In this phase, the animal was put in the light chamber. After entrance of the animal to the dark compartment with all the four feet (exploration time), the middle door was lowered and then an electrical shock (1 mA, 50 Hz, 2 seconds) was given to the floor of the dark chamber. Following 20 seconds of stay, the animal was removed from the apparatus.

#### 2.6.4. Retention test

The memory retention test was performed 24 hours after training. After placing the mice in the light compartment, the door was raised with a 10-second delay. Then, the time latency to enter the dark chamber was calculated, termed “Step Through Latency” (STL). This test was finished after the entrance of the animal to the dark chamber or if it stayed in the light compartment for 300 seconds.

### 2.7. Data analysis

One-way Analysis of Variance (ANOVA) followed by Tukey multiple comparison test was used for data analysis. The data are provided as Means±Standard Error of Mean (SEM) and P<0.05 was considered as the level of significance in all statistical analyses.

## 3. Results

### 3.1. Characterization of curcumin nanoparticles

**Figure 1.**
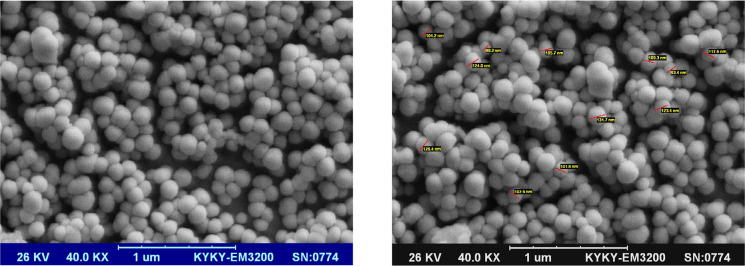
The SEM images of curcumin/BSA nanoparticles with different magnifications

The different magnifications of FESEM images of nanocurcumin are shown in Figure 1. The resulted spherical nanoparticles had a mean diameter of 110±14 nm.

### 3.2. The effect of nanocurcumin on scopolamine-induced memory retrieval deficit

**Figure 2.**
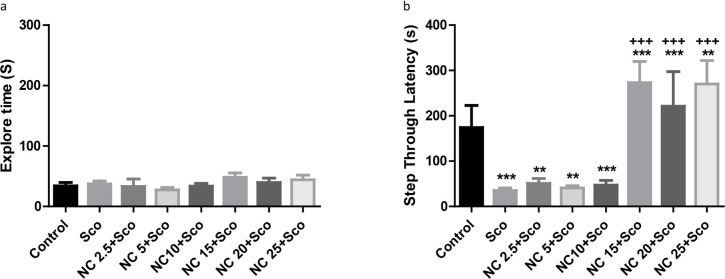
The results of the pilot study of nanocurcumin Figure 2 A shows the exploration time of animals during training session, which shows no significant difference between the groups; Figure 2 B depicts STL during memory retention trial. As it is observed, the doses of 15, 20, and 25 mg/kg nanocurcumin prevent scopolamine induced amnesia. Data are shown as Mean±SEM. ^**^ P<0.01; ^***^ P<0.001 indicate the difference between the control group and the others; ^+++^ P<0.001 represents the difference between scopolamine and scopolamine + nanocurcumin treated groups. Sco: Scopolamine; NC: Nanocurcumin

A pilot study was conducted to determine the effective doses of nanocurcumin against scopolamine induced amnesia. A wide range of doses (2.5, 5, 10, 15, 20, and 25 mg/kg/p.o.) was administered for 10 days in the groups of three mice. As shown in Figure 2, there was no difference between the groups in their exploration time during training. One-way ANOVA indicated a significant difference between the groups in their STL of retention time [F_(8, 16)_ =14.89, P<0.0001]. Post hoc Tukey test revealed that nanocurcumin in doses 15, 20, and 25 mg/kg prevented scopolamine-induced amnesia. Then, the doses of 15 and 20 were selected for the rest of the study.

**Figure 3.**
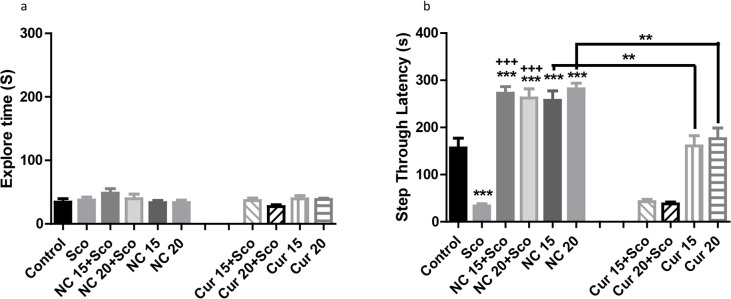
The effect of nanocurcumin/curcumin on memory Figure 3 A shows the exploration time of training session; and Figure 3 B illustrates STL of retention test. Data are illustrated as Mean±SEM; ^***^ P<0.001 shows the difference between the control group and the others; ^**^ P<0.01 represents the difference between nanocurcumin and curcumin treated mice; ^+++^ P<0.001 shows the difference between the groups of scopolamine and scopolamine+ nanocurcumin. Sco: Scopolamine; NC: Nanocurcumin; Cur: Curcumin

Figure 3 shows the effect of the doses of 15 and 20 mg/kg of nanocurcumin or natural curcumin with/without scopolamine in groups of eight rats. Although there was no difference in the initial exploration time on the day of training [Figure 3 A, F_(9, 70)_ =1.268, P=0.2696], the STLs of retention time were significantly different between the groups [Figure 3 B, F_(9, 70)_ =39.43, P<0.0001]. Post hoc test revealed that the doses of 15 and 20 mg nanocurcumin, prevented scopolamine-induced amnesia, while its equivalent doses of natural curcumin did not show such an effect. Interestingly, nanocurcumin -but not its equivalent doses of natural curcumin-by itself improved STL comparing with the control animals showing its ameliorative effect on memory. A comparison between nanocurcumin and its equivalent natural curcumin receiving groups revealed a significant difference (F_(3, 28)_=9.285, P=0.0002) and the results of Turkey test are depicted in Figure 3 B.

## 4. Discussion

Although the cognitive decline of patients with AD was previously supposed to originate from encoding failure (Hodges, Salmon, & Butters, 1990; Weintraub, Wicklund, & Salmon, 2012), a recent study in which the memory of amnesic transgenic mice was elicited through optogenetic activation of hippocampal memory engram cells revealed the significance of memory retrieval stage in AD (Roy et al., 2016).

The administration of drugs by oral route is considered as the most comfortable way (Rein et al., 2013; Scheepens, Tan, & Paxton, 2010). Meanwhile, for a substance such as curcumin, the issue of poor absorption and therefore low bioavailability exits (Li, Braiteh, & Kurzrock, 2005). Albumin is regarded as a safe carrier for curcumin due to its non-toxic and non-antigenic properties (Aniesrani Delfiya et al., 2016; Jithan et al., 2011).

The results of the current study indicated that BSA-based nanocurcumin prevented scopolamine induced memory retrieval deficit in passive avoidance task, while its equivalent doses of natural curcumin had no effect. Consistently, it was revealed that natural curcumin had no effect in clinical trials of AD (Baum et al., 2008; Ringman et al., 2012), while a more bioavailable form of curcumin (Longvida®) improved cognition in aged adults (Cox, Pipingas,& Scholey, 2015).

The efficiency of the nanoformulation of curcumin used in the current study might be resulted from its higher bioavailability. According to SEM results and the nano diameter of nanocurcumin particles, this curcumin formulation is supposed to have a greater bioavailability compared with that of natural curcumin. Additionally, albumin -present in this formulationhas endothelial transcytosis properties, which helps this formulation in entering the cells (John, Vogel, Tiruppathi, Malik, & Minshall, 2003; Minshall, Tiruppathi, Vogel, & Malik, 2002). In this regard, albumin-based nanocurcumin was previously reported to yield higher bioavailability in comparison to natural curcumin (Jithan et al., 2011; Kim et al., 2011).

The current study also revealed that nanocurcumin by itself had a boosting effect on memory retrieval. Then it is probable that the improvement observed in scopolamine-treated animals is resulted from the direct effect of nanocurcumin on memory performance. Although it is reported that curcumin moderates the acetylcholinesterase activity in the hippocampus (Oz, Nurullahoglu Atalik, Yerlikaya, & Demir, 2015), some experiments show that the memory enhancing effect of curcumin against scopolamine is not accompanied by acetylcho-linesterase inhibition (Ahmed & Gilani, 2009).

Scopolamine-induced memory impairment is accompanied by oxidative stress (El-Khadragy, Al-Olayan, & Abdel Moneim, 2014). The ameliorative effect of curcumin against scopolamine-induced memory disruption might be related to its antioxidant effect, since it is observed that curcumin is at least 10 times more active, as an antioxidant, than vitamin E (Khopde et al., 2000).

In conclusion, the finding of the current study indicated that a BSA based nanocurcumin in doses of 15 and 20 mg/kg -the doses which natural curcumin has no effect-prevented scopolamine-induced memory retrieval deficit. Due to the low bioavailability of curcumin and that some epidemiological studies suggest its beneficial effects using nanotechnology the current study might be helpful to clarify the effects of curcumin on memory.

## Ethical Considerations

### Compliance with ethical guidelines

All experiments were approved by the Ethics Committee for Animal Experiments of Shiraz University of Medical Sciences and the animal experiments were conducted in accordance with the NIH Guide for the Care and Use of Laboratory Animals. The mice were randomly allocated to groups of eight for the main work and groups of three for the initial pilot study.
